# Localized Spin Textures Stabilized by Geometry‐Induced Strain in 2D Magnet Fe_3_GeTe_2_

**DOI:** 10.1002/adma.202506279

**Published:** 2025-06-18

**Authors:** Yuhan Sun, Max T. Birch, Simone Finizio, Lukas Powalla, Sayooj Satheesh, Tim Priessnitz, Eberhard Göring, Ernst Knöckl, Christoph Kastl, Alexander Holleitner, Klaus Kern, Markus Weigand, Sebastian Wintz, Marko Burghard

**Affiliations:** ^1^ Max Planck Institute for Solid State Research Heisenbergstrasse 1 70569 Stuttgart Germany; ^2^ RIKEN Center for Emergent Matter Science Wako 351‐0198 Japan; ^3^ Swiss Light Source Paul Scherrer Institute (PSI) Villigen 5232 Switzerland; ^4^ Helmholtz‐Zentrum Berlin für Materialien und Energie GmbH Institut Nanospektroskopie 12489 Berlin Germany; ^5^ Walter Schottky Institute and Physics Department Technical University of Munich 85748 Garching Germany

**Keywords:** 2D magnets, Fe_3_GeTe_2_, higher order topological spin textures, skyrmions, strain engineering

## Abstract

Strain engineering promises to enable manipulation and control of the properties of exfoliated flakes of 2D van der Waals (vdW) ferromagnets for spintronic applications. However, while previous studies of strain effects have focused on global properties, the impact on local magnetic spin textures remains unexplored. Here, manipulation of magnetism in the 2D ferromagnet Fe_3_GeTe_2_ (FGT) is demonstrated using geometry‐induced strain. Employing scanning transmission X‐ray microscopy (STXM), the effects of spatially varying strain profiles on the magnetic order of FGT sheets stamped onto micropillar arrays are directly visualized. It is found that the in‐plane strain components, with magnitudes <0.5%, locally elevate the Curie temperature of FGT by 10 K, stabilizing magnetic domains near the pillar corners. These domains include skyrmions and higher‐order topological spin textures such as skyrmioniums and skyrmion bags. The possibility to locally seed and control topological spin textures via strain opens new avenues for future spin‐based information technologies.

## Graphical abstract

Strain engineering in 2D van der Waals ferromagnet Fe₃GeTe₂ is studied using scanning transmission X‐ray microscopy. Spatially varying strain from micropillar arrays induced local 10 K Curie temperature increases, stabilizing magnetic domains including skyrmions and skyrmion bags near pillar corners. This strain‐control of topological spin textures offers new avenues for spintronic technologies.

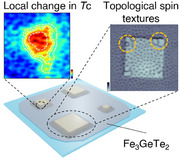

## 1 Introduction

Two‐dimensional (2D) van der Waals (vdW) magnets and their heterostructures have emerged as a new platform for exploring fundamental physics and future device applications.^[^ ^1^, ^2^ ^]^ Notably, their layered structure renders them more amenable to control via external stimuli, as compared to their thin film counterparts, for instance via electric fields.^[^ ^3^, ^4^ ^]^ As 2D materials in general are capable of undergoing substantial elastic deformations, a promising approach is strain engineering, which allows for significant modifications to their electronic, optical, and magnetic properties.^[^ ^5^ ^]^ This enables the exploration of a wide range of physical phenomena,^[^ ^6^ ^]^ such as by the introduction of pseudo‐magnetic fields in bilayer graphene,^[^ ^7^ ^]^ a change of transition temperature of conventional and unconventional 2D superconductors,^[^ ^8^ ^]^ the introduction of superconducting diodicity,^[^ ^9^ ^]^ as well as tuning of the optical emission of transition metal dichalcogenides (TMDCs),^[^ ^10^ ^]^ or the realization or enhancement of photovoltaic effects by strain‐induced inversion symmetry‐breaking.^[^ ^11^, ^12^ ^]^ With respect to 2D magnets, it has been documented that strain can induce a phase transition between antiferromagnetic and ferromagnetic states in CrI_3_ and CrSBr,^[^ ^13^, ^14^ ^]^ influence the antiferromagnetic order in FePS_3_ and NiPS_3_,^[^ ^15^ ^]^ and enhance the Curie temperature *T*_C_ of Cr_2_GeTe_6_.^[^ ^16^ ^]^

Among 2D magnets, metallic Fe_3_GeTe_2_ (FGT) has attracted considerable attention due to its intrinsic itinerant ferromagnetism, which persists down to the monolayer limit, and its relatively high Curie temperature (*T*_C_) of up to 220 K in the bulk. There are now numerous reports of topological spin textures such as skyrmions,^[^ ^17^, ^18^, ^19^, ^20^, ^21^, ^22^, ^23^, ^24^, ^25^, ^26^, ^27^, ^28^, ^29^, ^30^ ^]^ as well as skyrmion bubbles and skyrmion bags, forming in thin samples of the material.^[^ ^31^, ^32^, ^33^ ^]^ It has been experimentally demonstrated that applying tensile strain to FGT can modulate the coercive field *H*_c_, increase *T*_C_, and induce magnetic phase transitions.^[^ ^34^ ^]^ In addition, it has been reported that applying a voltage to a vdW heterostructure device made of FGT and ferroelectric In_2_Se_3_ significantly decreases the coercive field of FGT.^[^ ^35^ ^]^ This effect has been attributed to in‐plane tensile strain induced by the voltage, which reduces the magnetocrystalline anisotropy of FGT. Thus far, however, these effects have been probed mainly by indirect measurements like charge transport,^[^ ^5^ ^]^ and have thus been limited to the global scale and the most common types of strain (i.e., uniaxial or biaxial strain). Accordingly, the influence of localized strain on the formation and spatial distribution of the magnetic domains and topological spin textures, such as skyrmions and higher‐order skyrmionium and skyrmion bags, remains essentially unexplored. Here, we address this gap by using soft X‐ray microscopy to directly visualize and map the spatial evolution of magnetic properties and spin textures in exfoliated FGT sheets that are subjected to localized strain via an underlying micropillar.

## 2 Results and Discussion

### 2.1 Bulk Magnetic Characterization

**Figure 1:**
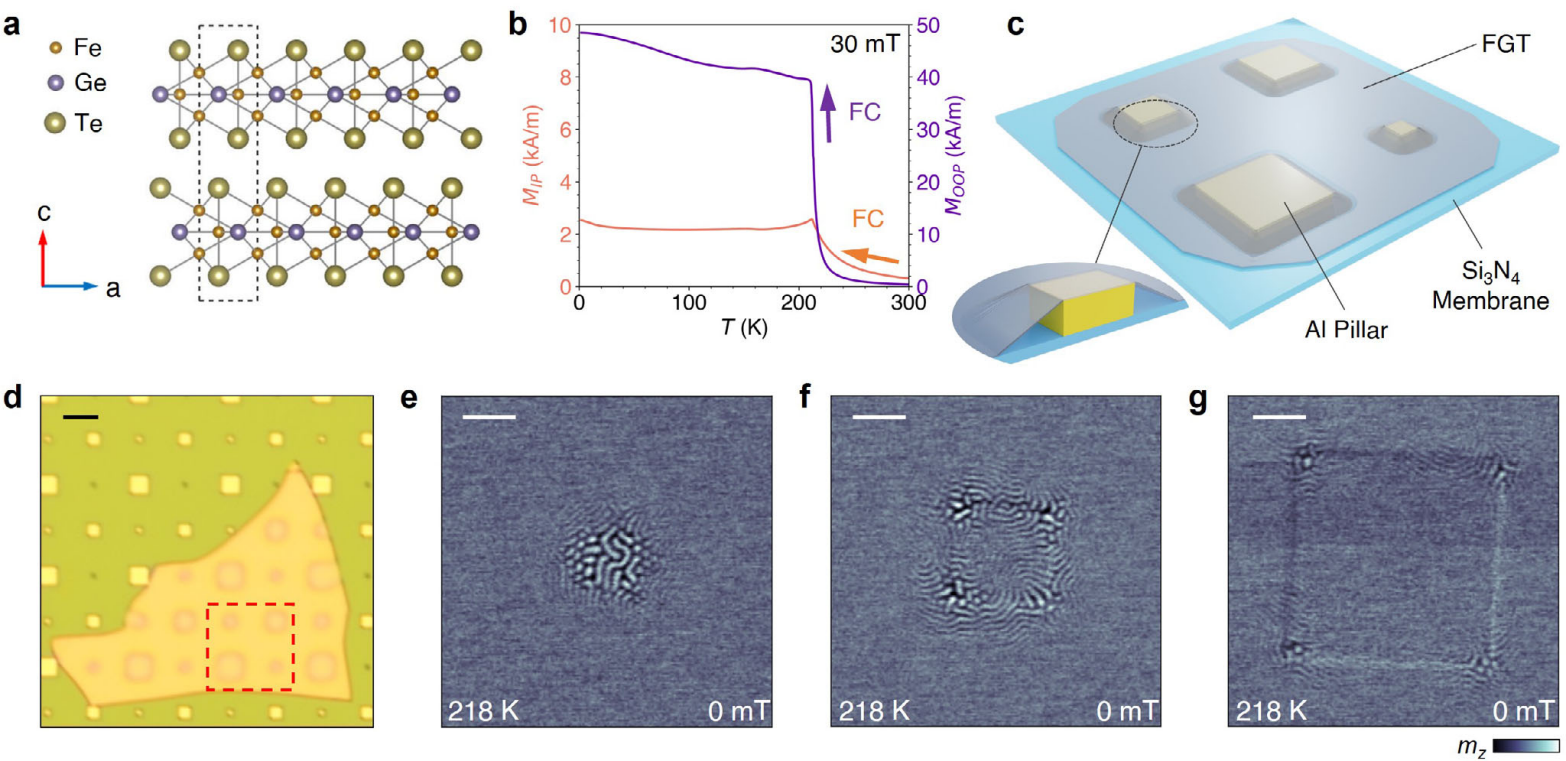
Straining of the Fe_3_GeTe_2_ (FGT) flake by a micropillar array underneath. a) Side view of the FGT crystal structure. b) Magnetization versus temperature with a 30 mT magnetic field applied in both out‐of‐plane (purple) and in‐plane (orange) directions of the bulk FGT crystal lattice. c) Schematic of the strained FGT device. First, an array of Al pillars (side lengths: 1, 2, 4, and 6 µm) was prefabricated on a Si₃N₄ membrane, followed by stamping of a uniform FGT flake onto the pillar structure. d) Optical micrograph of the strained FGT flake on the Si₃N₄ membrane with Al pillars. The region of interest (ROI) is indicated for subsequent STXM images. Scale bar: 10 µm. e‐g) STXM images of magnetic texture in the ROI at 0 mT and 218 K. Al pillar side lengths are 1, 2, and 4 µm, respectively. Scale bar: 1 µm.

FGT has a layered hexagonal crystal structure, where the Fe_3_Ge heterometallic slabs are sandwiched by Te layers (**Figure** **1**a).^[^ ^36^ ^]^ It exhibits a large uniaxial magnetocrystalline anisotropy with the easy axis along the c axis,^[^ ^37^ ^]^ while the *T*_C_ of bulk crystals ranges from 150 to 220 K depending on the Fe deficiency.^[^ ^36^ ^]^ SQUID magnetization measurements as a function of temperature with magnetic field applied along both the out‐of‐plane (H||c) and in‐plane (⊥*c*) directions of our bulk FGT crystals under an applied field of 30 mT are presented in Figure 1b. The data yield a bulk *T*_C_ of 212 K, and confirm the uniaxial magnetocrystalline anisotropy along the *c*‐direction (see Figure S1, Supporting Information, for more magnetometry data).

### 2.2 Effect of Local Strain on Magnetic Phases

To explore possible local strain effects, the bulk crystal was mechanically exfoliated and the obtained FGT flakes were stamped onto a micropillar array prepared on a 200 nm thick Si_3_N_4_ membrane, as schematically illustrated in Figure 1c. The square‐shaped micropillars are made of aluminum with a height of 245 nm (see Experimental Section), which is thin enough to be translucent to soft X‐rays at the Fe *L*_3_ absorption edge, enabling our transmission x‐ray imaging measurements. Similar structural arrays have previously been used to introduce mechanical strain into graphene or TMDC sheets.^[^ ^38^, ^39^ ^]^ As visible from the optical micrograph in Figure 1d, the FGT sheet naturally drapes over the pillars due to attractive vdW interactions with the substrate, leading to local regions of strain at the pillar sites. Importantly, the geometry of the pillars creates a localized strain profile, depending on the size of the underlying structures and the mechanical properties of the FGT flake.

To investigate the local influence of the mechanical strain on the FGT sheet, we utilized scanning transmission x‐ray microscopy (STXM) with high spatial resolution of 25 nm, which exploits the x‐ray magnetic circular dichroism (XMCD) to obtain the local out‐of‐plane component of the magnetization *m*_z_ (see Experimental Section)_._^[^ ^40^ ^]^ The region of interest, highlighted by the dashed red frame in Figure 1d, includes four square pillars of varying sizes, with side lengths of 1, 2, 4, and 6 µm. The STXM images shown in Figure 1e–g illustrate the formation of stripe domains and skyrmions concentrated around each structure at 218 K – above the *T*_C_ of the original bulk crystal. The surrounding FGT flake shows no magnetic ordering. Each panel is a subtraction of images acquired for left and right circular X‐ray polarization, which mostly eliminates the structural contrast, and leaves the magnetic contrast. As the size of the pillars increases, these magnetic textures become more localized at the corners of the micropillars, which appears to indicate that these areas experience the strongest local strain (see Figure S2, Supporting Information, for the overview STXM image of the investigated region marked in Figure 1d).

**Figure 2:**
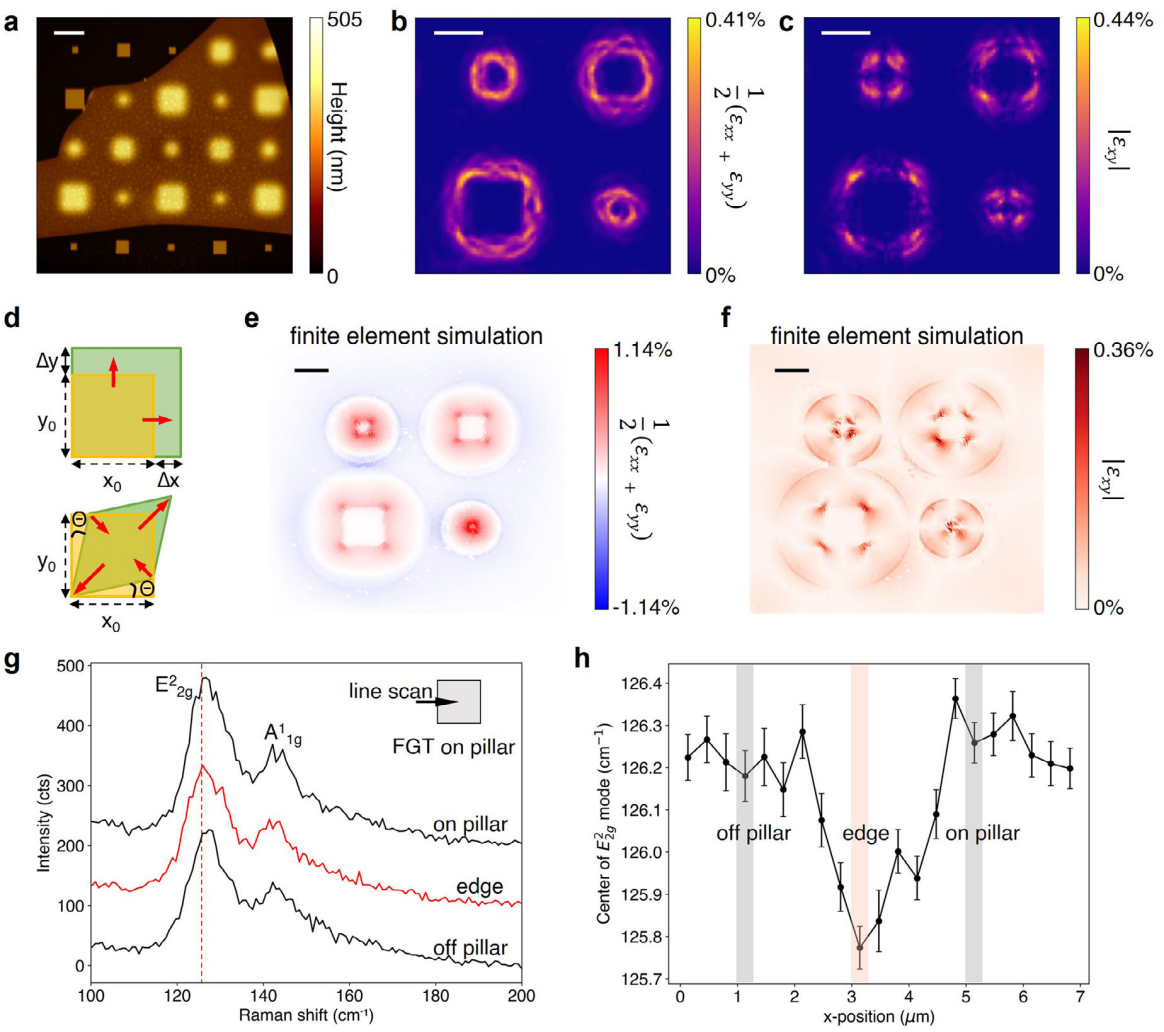
Local strain distribution in the FGT flake. a) Tapping mode atomic force microscopy image of the FGT flake on the micropillar array. From the corresponding section profiles, the height of the Al pillars was determined to be 245 nm, and the thickness of the FGT flake to be 155 nm. Scale bar: 10 µm. b,c) Maps of the in‐plane tensile strain b) and the shear strain c) calculated from the topographic AFM data in a). Scale bar: 5 µm. d) Schematic of lattice distortions under in‐plane tensile strain (top sketch) and shear strain (bottom sketch). e,f) Maps of the simulated in‐plane normal strain distribution e) and the simulated in‐plane shear strain f). Scale bar: 5 µm. g) Raman spectra of FGT measured across an underlying pillar with 4 µm side length. The red shift of the modes can be discerned already in the bare spectrum (dashed line as a guide to the eye). h) Fitted position of the E^2^_2g_ mode in FGT when scanning across the pillar edge. The error bars are the 1σ‐confidence intervals of the parameter as evaluated by the fit routine.

We estimated the induced local strain distribution in the FGT flake from atomic force microscopy (AFM) height profiles using the strain tensor defined as:

(1) $$\varepsilon_{ij}\;\left(r\right)=\frac{1}{2}\;\left(\partial_{i}u_{j}\left(r\right)+\partial_{j}u_{i}\left(r\right)+\partial_{i}h\left(r\right)\partial_{j}h\left(r\right)\right)$$

where *u*(***r***) and *h*(***r***) represent the in‐plane and out‐of‐plane deformation fields, respectively. We rely upon the assumption that the in‐plane deformation field is significantly smaller than the out‐of‐plane deformation field.^[^ ^39^ ^]^ From the AFM image of the flake on the micropillar array (**Figure** **2**a), its thickness was determined to be 155 nm. The map in Figure 2b reveals a biaxial tensile strain, calculated as (*ɛ*_*xx*_ + *ɛ*_*yy*_)/2, of ≈0.4% which is almost uniformly distributed along the edges of the pillars. This uniform distribution does not align well with the observed magnetic behavior of the flake, where the most significant effects appear concentrated at the micropillar corners. In comparison, the shear strain *ɛ*_*xy*_ distribution, shown in Figure 2c, reaches a similar magnitude but is more localized at the corners, in accordance with the experimental observation in Figure 1f,g (for further strain maps see Figure S3, Supporting Information). The deformations associated with tensile and shear strain are sketched in Figure 2d. The formation of sizable shear strain is likely enabled by the moderate bending of the flake over the pillars, in contrast to more flexible 2D sheets like graphene which more closely conform to the pillar profile, whereupon shear strain is partially released.^[^ ^39^ ^]^ This is confirmed by our finite element simulations of a model flake stretched over a pillar, shown in Figure 2e and f, which substantiate the strong localization of both tensile strains at the pillar edges and shear strain at the pillar corners, while the top surface of the pillar is almost strain‐free (more details can be found in Figure S4, Supporting Information). Furthermore, direct experimental proof for the local tensile strain along the pillar edges could be obtained by Raman microscopy. Specifically, the E^2^_2g_ mode at ≈126 cm^−1^ mode in FGT experiences a red shift at the pillar edges, as evidenced by Figure 2g and h. Based upon previous observations on strained FGT,^[^ ^41^ ^]^ one estimates a tensile strain of ≈0.5% from the magnitude of the redshift (≈0.6 cm^−1^) (Figure S5, Supporting Information).

### 2.3 Local Change of Magnetic Ordering

**Figure 3:**
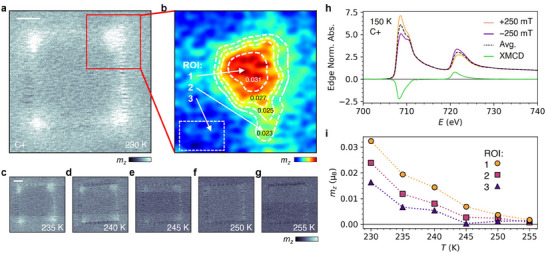
Spatially resolved temperature‐induced change of magnetization. a) X‐ray micrograph of the FGT flake on top of the pillar with side length of 2 µm, acquired at C+ and 230 K. Scale bar: 500 nm. b) Zoomed‐in image of magnetization at one corner from a). White contours show lines of constant *m_z_*. c–g) STXM images of FGT at C+ displayed at five different temperatures. All X‐ray micrographs are a subtraction of images acquired with +250 mT and −250 mT. Scale bar: 500 nm. The contrast difference in the background is caused by step‐wise changes in X‐ray intensity during scanning, caused by storage ring top‐up events. h) X‐ray absorption spectra of the FGT flake at 150 K with left‐hand circular polarization under applied fields of ±250 mT. The difference between the spectra yields the XMCD signal. i) Out‐of‐plane magnetization (*m_z_*) as a function of temperature at the regions of interest (ROI) defined in b). *m*_z_ was calculated by averaging the magnetic contrast within each region and converting it to spin moment using the sum rules analysis.

In order to elucidate the effect of the local strain on the magnetic properties in greater detail, we acquired a series of STXM images (**Figure** **3**a–g) of the 2 µm nanopillar acquired for right circular X‐ray polarization at temperatures between 230 and 255 K, above *T*_C_. In Figure 3a, the brighter contrast at the corners indicates a larger out‐of‐plane magnetization *m*_z_ – our STXM technique is sensitive enough to detect the small magnetization induced by the applied magnetic field in this paramagnetic region. To quantify the observations, X‐ray absorption spectra of the FGT flake were acquired at 150 K for both positive and negative applied fields (Figure 3h), revealing a strong XMCD signal. Via the sum rules analysis,^[^ ^42^, ^43^ ^]^ we determined the spin and orbital contributions to the magnetic moment to be 1.11 µ_B_ and 0.18 µ_B_, respectively (see Figure S6, Supporting Information, for details). With this XMCD calibration, we show a closer view at one corner of the pillar, revealing significant spatial variation in *m*_z_ due to the strain. The plotted *m*_z_ contours show a reduction of *m_z_* by 75% over ≈50 nm, demonstrating the significant effect of the spatially varying strain profile. As the temperature increases, the field‐induced magnetization of the unstrained regions approaches zero at 245 K, while at the corners this does not occur until 255 K, whereupon all magnetic contrast is lost (Figure 3g). Thus, from the temperature dependence of *m*_z_ for the three different ROI (Figure 3i), it can be deduced that at the corners the temperature of vanishing magnetization, and as will be confirmed later, *T*_C_ itself, increases by 10 K compared to the less strained regions of the flake. Overall, it is plausible to assume that for the observed local change in magnetic ordering, both the tensile and shear strain contributions are essential, albeit the latter may cause more pronounced symmetry breaking of the crystal lattice which is known to significantly impact the magnetic properties of materials.^[^ ^44^ ^]^ As an alternative, the observed changes may be due to the deformation of the FGT flake at the corners, leading to a compression of the crystal lattice along the c‐axis. However, this effect can be ruled out as the main cause of the observed change in magnetic properties, because previous experiments have shown that *T*_C_ decreases with increasing pressure along the *c*‐axis direction.^[^ ^45^, ^46^ ^]^ This decrease has been attributed to the diminished 3*d* electron correlations which are caused by the shortening of Fe‐Fe distance.^[^ ^37^ ^]^ Moreover, the strain effect does not exhibit a threshold behavior but rather scales continuously with respect to local strain magnitude (see Figure S7, Supporting Information, for details). This observation is consistent with previous experimental and theoretical studies reporting an approximately linear relationship between *T*_C_​ and strain.^[^ ^5^, ^34^, ^47^ ^]^

The observed enhancement of *T*_C_ due to tensile strain can be attributed to modifications in both the magnetic exchange interaction and magnetocrystalline anisotropy. In atomically thin FGT flakes, magnetocrystalline anisotropy is crucial for sustaining ferromagnetism by suppressing fluctuations that could disrupt long‐range order, thus affecting *T*_C_​, while the spin exchange couplings are used to estimate *T*_C_​ in the bulk limit of FGT.^[^ ^5^ ^]^ The changes in *T*_C_​ originate from the interplay between direct exchange interactions from Fe─Fe bonds and super‐exchange interactions from Fe─Te─Fe bonds. The direct exchange, involving electron hopping between nearest‐neighbor Fe sites via 3*d* orbitals, typically exhibits antiferromagnetic (AFM) ordering. Conversely, the indirect exchange, mediated through nonmagnetic ions like Ge or Te, can result in either ferromagnetic (FM) or AFM ordering, depending on the bond angle, incorporating both super‐ and double‐exchange interactions.^[^ ^48^ ^]^ According to the Goodenough‐Kanamori‐Anderson rules, an AFM coupling occurs when the magnetic‐ion‐magnetic angle is 180°, while FM coupling is favored when the bond angle nears 90°.^[^ ^37^ ^]^ Under tensile strain, the Fe─Fe bond length increases, weakening the direct‐exchange interaction and the associated AFM coupling. Simultaneously, the Fe─Te─Fe bond angle approaches 90°, enhancing the FM coupling favored by indirect exchange. This overall effect leads to an increase in *T*_C_​ under tensile strain, as the FM interactions become more dominant. Compared to tensile strain, much less is known about the influence of shear strain on the magnetic properties of FGT and 2D magnets in general. Although Monte Carlo simulations indicate that pure shear strain (2–10%) minimally affects the *T*_C_ of 2D magnetic materials, these findings likely oversimplify the underlying physics.^[^ ^49^ ^]^ Importantly, shear strain can reconfigure the angular relationships between magnetic exchange paths, a mechanism requiring rigorous examination through advanced DFT calculations to fully elucidate strain‐magnetism coupling in these systems.

### 2.4 Magnetic Phase Diagrams

We further explored the influence of the local strain on the spin texture formation as a function of temperature and applied magnetic field, following a field‐sweeping (FS) protocol, where images were acquired with increasing *B*‐field after the flake was initialized in the uniformly magnetized state at −250 mT. In general, the domain structures resemble those of an unstrained FGT flake: below 150 K the flake exhibits uniform switching behavior, with no domain nucleation; >150 K and <210 K stripe domains nucleate close to zero fields, while finally above 210 K skyrmion bubble formation is also observed.^[^ ^40^, ^50^ ^]^ In general, the characteristic domain size decreased at higher temperatures. However, the presence of the microstructure affected each regime in a different manner, and to varying degrees.

**Figure 4:**
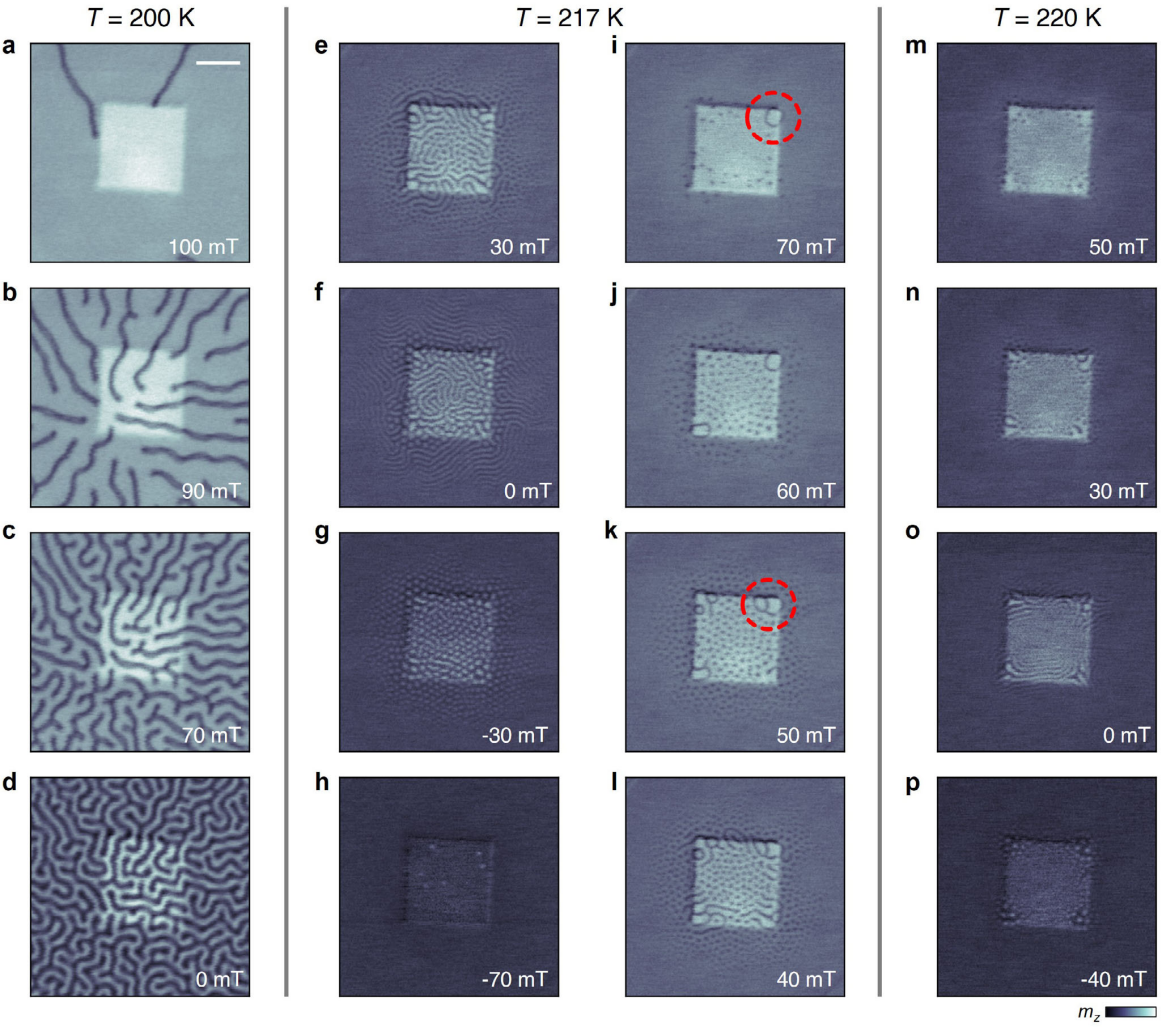
Real‐space imaging of spin textures in FGT. a–p) X‐ray micrographs of the Fe_3_GeTe_2_ flake on top of the Al pillar with a 2 µm side length, measured during field sweeps from −250 to 250 mT at temperatures of 200 (a–d), 217 (e–l), and 220 K (m–p). Each image's magnetic contrast has been normalized to emphasize magnetic structures. The grayscale intensity corresponds to the out‐of‐plane magnetization (*m_z_*). Red dashed circles highlight the emergence of a skyrmionium i) and a skyrmion bag k). Scale bar: 1 µm.

Images acquired at selected temperatures are compared in **Figure** **4** (further data shown in Figures S8 and S9, Supporting Information). The contrast scale is normalized by dividing each image by its corresponding saturated state at −250 mT. At 200 K (Figure 4a–d), the FGT flake exhibits labyrinthine stripe domains for moderate magnetic fields around zero field. At this temperature, we observed the formation of a radially ordered domain state centered on the pillar (Figure 4b). This structure is likely formed due to the domains aligning along the directions of maximum strain gradient to minimize energy. At slightly higher temperature (217 K), as presented in Figure 4e–l, the magnetic textures become more complex, with various spin textures coexisting. At higher magnetic fields, the stripe domains are broken up into individual skyrmions, which appear to be preferentially stabilized at the corners of the microstructures. A further remarkable observation is the formation of skyrmionium (Figure 4i), and skyrmion bags (Figure 4k), at the micropillar corners. At increased temperature (220 K), spin texture formation is localized at the micropillar corners, while the surrounding flake exhibits only uniform magnetization (Figure 4m–p), consistent with the increase of *T*_C_ observed in Figure 3.

The above results reveal that the influence of the strain depends crucially on the temperature of the sample, and we speculate that this is due to the interplay of the characteristic domain size with the length scale of the localized strain variation. For example, in the 200 K data, the characteristic domain size appears to be too large to observe localized spin texture variation via the local strain at the micropillar corners. Instead, we suggest it is the larger feature of the flake bent over the pillar that gives rise to the radial domain ordering. At 217 K, the domain size becomes comparable to or smaller than the in‐plane strain profile at the corners, giving rise to the preferential stabilization of topological spin textures, including skyrmionium and skyrmion bags. In past work on an unstrained FGT flake, we showed that these higher‐order topological states could typically not be nucleated via a field‐sweep procedure, due to the mutual repulsion of neighboring domain walls, which must be connected to form the loop‐like states,^[^ ^51^ ^]^ and this energy barrier could only be overcome close to *T*_C_ during a zero field cooling procedure. However, in the present case, it appears that the strain profile around the micropillar corners opens the energetic pathway for the formation of these complex magnetic orderings at temperatures below *T*_C_. The formation of the skyrmionium states was also observed at the corners of pillars with side lengths of 1, 4, and 6 µm (see Figures S10 and S11, Supporting Information).

**Figure 5:**
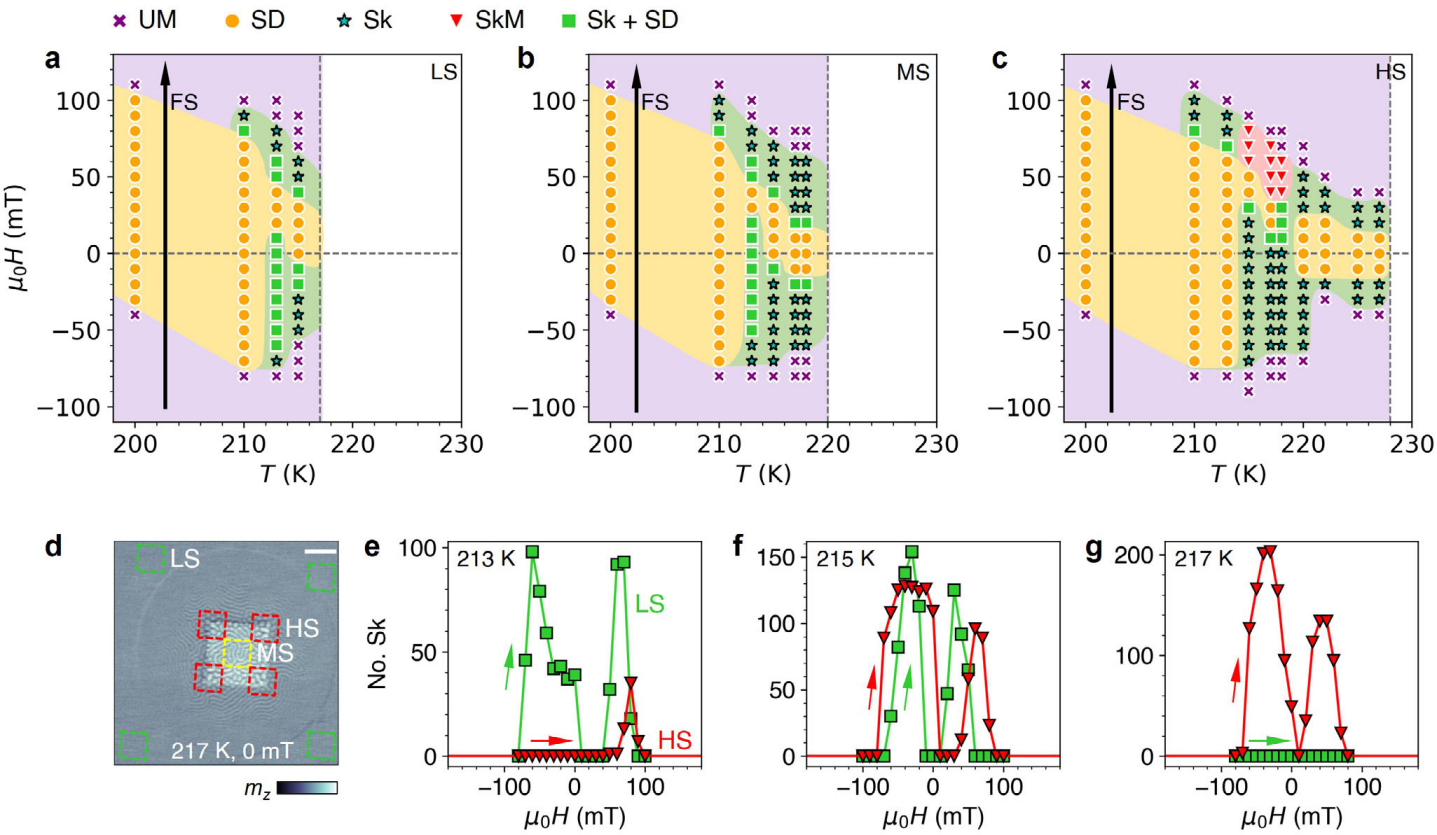
Strain‐dependent magnetic phase diagrams and number of skyrmions. a–c) Magnetic phase diagrams of the FGT flake are shown for three regions with varying strain: low strain a), medium strain b), and high strain c). These diagrams are derived from imaging the flake using a field sweep protocol as a function of temperature and applied magnetic field. Arrows indicate the measurement path (field sweep). The phase diagrams are labeled according to the observation of uniform magnetization (UM), stripe domains (SD), skyrmions (Sk), and skyrmionium (SkM) states. d) X‐ray micrograph of the FGT flake at 217 K and 0 mT. The colored frames mark representative regions of low strain (green), medium strain (yellow), and high strain (red). Scale bar: 1 µm. e–g) Number of skyrmions as a function of applied field in low strain (green) and high strain (red) regions at temperatures of 213 e), 215 f), and 217 K g). Arrows indicate the measurement direction.

The imaging experiments are summarized in magnetic phase diagrams for three areas of the FGT flake, displayed in **Figure** **5**a–c, corresponding to low strain (LS), medium strain (MS), and high strain (HS) regions, which are defined by the colored boxes in Figure 5d. The phase diagrams are labeled according to the observation of uniform magnetization (UM), stripe domains (SD), skyrmions (Sk), and skyrmionium (SkM) states. Points where the stripe domain and skyrmion states coexisted were included in the skyrmion region of the phase diagrams for clarity. In general, the phase diagrams are asymmetric ≈0 mT due to the direction of the FS measurement protocol.^[^ ^40^ ^]^ In the low‐strain region (Figure 5a), the skyrmion state is only observed in a small region of applied fields and temperatures close to *T*_C_, in accordance with previous reports on exfoliated FGT.^[^ ^40^ ^]^ We take the temperature corresponding to the disappearance of discernible spin textures as an approximation for *T*_C_ in our system, and thus *T*_C_ for the low‐ and high‐strained FGT regions is determined from the corresponding phase diagrams to be 217 and 228 K, respectively. This strain‐induced increase of *T*_C_ by at least 10 K is in good agreement with the temperature differences deduced from Figure 3i.

On this basis, as the local strain increases, the Sk state persists at higher temperatures and across a wider range of applied fields, suggesting that local strain energetically favors skyrmion nucleation. This is illustrated in Figure 5e–g, which displays the number of skyrmions as a function of applied field in the LS (green) and HS (red) regions at three representative temperatures, with the area of the LS region counted being equal to that of the HS regions. While at 213 K (Figure 5e), the skyrmions predominantly localize in the low strain region, at 215 K (Figure 5f) there are comparable skyrmion numbers in both low and high strain regions, and at 217 K (Figure 5g) skyrmions exist only in the HS region. This trend reflects a strain‐induced shift of the skyrmion pocket. Furthermore, as shown in Figure 5d, the average diameter of the skyrmions is larger in the HS regions. Finally, in the HS region, skyrmionium states emerge within a narrow range of temperatures and applied fields.

## 3 Conclusion 

In summary, real‐space magnetic imaging of an FGT flake on a micropillar array demonstrated that geometry‐induced strain profiles are effective for locally tailoring the magnetic properties of a 2D magnetic sheet. The local increase of *T*_C_ of our sample appears to be attributable to the in‐plane strain at the corners of the pillars. The ability to locally tune the magnetic properties through nanopatterning opens up new possibilities for designing spintronic devices with spatially varying magnetic properties, implemented, for instance, as nucleation points for skyrmions, as well as higher‐order topological spin textures, such as skyrmionium and skyrmion bags.

Although this work focuses on the imaging of strain‐induced spin textures, the underlying mechanism offers promising avenues for integration into spintronic devices. In particular, strain engineering could be combined with gate‐tunable substrates, such as piezoelectrics or ferroelectrics, to enable dynamic and localized control of spin configurations. To this end, the required gate insulator can be integrated into vdW heterostructures. Furthermore, electrical readout could be realized via integration with magnetoresistive elements (e.g., spin valves or MTJs), offering a route toward practical, strain‐controlled logic or memory components.

## 4 Experimental section

### Sample Preparation and Characterization

The FGT single crystal was synthesized using a chemical vapor transport technique. The crystal composition was confirmed by energy‐dispersive X‐ray spectroscopy (EDX) measurements using a Zeiss scanning electron microscope (SEM) Gemini 500, equipped with a Bruker XFlash 6–60 detector. Magnetometry measurements were conducted using a Quantum Design MPMS3 vibrating sample magnetometer. The bulk FGT crystal was aligned along the relevant crystal axis and affixed to a quartz glass rod with GE varnish. The micropillar array was fabricated by means of standard e‐beam lithography, utilizing a poly (methyl methacrylate) double‐layer resist. Following e‐beam exposure and development, ≈250 nm of aluminum was thermally evaporated without the use of an adhesion promoter, after which lift‐off was conducted. The strained FGT flake sample for the STXM experiments was prepared using an all‐dry viscoelastic transfer method. The initial stage of the process involved the mechanical cleaving and exfoliation of the FGT bulk crystal onto a PDMS stamp. Subsequently, a regular FGT flake with a thickness exceeding 100 nm, as estimated from the optical contrast, was selected and stamped onto the Al micropillar array on the membrane substrate. The entire process was conducted under ambient conditions. Immediately after finalizing the sample, it was sealed under vacuum to minimize surface oxidation of the FGT. In a previous study utilizing FGT flakes in this manner, it was found that the surface oxide layer was ≈7 nm in thickness.^[^ ^40^ ^]^ To determine the thickness of the strained FGT flake and obtain height profiles for the strain calculations, measurements were performed on the strained FGT flake using a Bruker Dimension Icon atomic force microscope (tapping mode). It was observed that flakes around 150 nm in thickness offer an optimal balance for the study, providing sufficient structural integrity and strain without exceeding the soft X‐ray penetration depth (see Figure S12, Supporting Information, for details).

### Scanning Transmission X‐ray Microscopy

STXM measurements were performed with the MAXYMUS instrument at the BESSY II electron storage ring operated by the Helmholtz‐Zentrum Berlin für Materialien und Energie. After mounting the sample in the microscope, cooling was achieved by using a helium cryostat, while the magnetic field was regulated by changing the configuration of four permanent magnets. The X‐ray beam was focused to a spot size of 25 nm using a Fresnel zone plate and an order selection aperture, which determined the approximate spatial resolution. Using piezoelectric motors, the sample was scanned pixel by pixel through the focused X‐ray beam of 708 eV nominal photon energy. By utilizing the XMCD effect at the X‐ray resonance energy at the Fe‐L_3_ edge, the transmission of the sample was quantified at each point to create an image of the non‐magnetic and magnetic contrast, with the photons counted by an avalanche photodiode. The magnetic signal was proportional to the out‐of‐plane magnetization *m_z_*. Images of the magnetic domain structures in Figure 4 were acquired using a single circular X‐ray polarization, while for the STXM images of Figure 1, and the temperature dependent measurements in Figure 3, both X‐ray polarisations were acquired, and a subtraction was made to acquire the pure XMCD contrast.

### Raman Microscopy

To independently verify the presence of strain in the FGT film on micropillars and to estimate its magnitude, Raman spectroscopy was performed. All measurements were carried out with a WITec Alpha Raman microscope (532 nm cw‐excitation, 55 µW laser power measured at the back aperture of the objective, 50x objective with NA = 0.75, 1800 lines/mm grating). The error bars are the 1σ‐confidence intervals of the parameter as evaluated by the fit routine.

### Finite Element Simulations

To obtain the full mechanical strain information for the FGT sheet, a numerical simulation of the ROI based on the finite element method (FEM) was performed using COMSOL Multiphysics 6.1.^[^ ^52^ ^]^ The mechanical properties of FGT are estimated based on values reported for other common 2D materials,^[^ ^53^ ^]^ specifically using density ρ  =  7300 kg m^−3^, Young's modulus *E*  =  265 GPa and Poisson's ratio 0.25. The pillars have the same nominal dimensions as in the experiments, i.e., a height of 245 nm and varying edge width *w_pillar_* of 1, 2, 4, and 6 µm, respectively. To match the experimentally observed topography (see Figure 2a in the main text) of the FGT flake, the flake is enforced to touch the Si_3_N_4_ membrane outside of a region around a pillar given by a circle of radius *r* = *w_pillar_* + 3.335 µm centered on each pillar. It was noted that this assumption results in artifacts at the radial edges where contact to the membrane is enforced.

## Conflict of Interest

The authors declare no conflict of interest.

## Author Contributions

Y.S. and M.T.B. contributed equally to this work. Y.S., M.T.B., and M.B. conceived the project; Y.S. fabricated the micropillars, performed the exfoliation and stamping procedures, and acquired the AFM images; Y.S., M.T.B., S.F., M.W., and S.W. performed the X‐ray microscopy imaging. E.K. and C.K. carried out the Raman microscopy experiments; T.P. performed the COMSOL simulations; E.G. performed the SQUID measurements; Y.S. and M.T.B. analyzed the data. The manuscript was written by Y.S., M.T.B., and M.B., with input from all authors. All authors discussed the results and provided feedback on the manuscript.

## Supporting information



Supporting Information

## Data Availability

The data that support the findings of this study are available from the corresponding author upon reasonable request.
