# Supplementary material for: Localized Spin Textures Stabilized by Geometry‐Induced Strain in 2D Magnet Fe3GeTe2
Source: Adv Mater. 2025 Jun 18;37(37):2506279. doi: 10.1002/adma.202506279 (PMC12447047; doi:10.1002/adma.202506279)
Supplement: Supplementary file 1 — Supporting Information [file ADMA-37-2506279-s001.docx]

**Supporting Information**

**Localized spin textures stabilized by geometry-induced strain
in 2D magnet Fe_3_GeTe_2_**

*Yuhan Sun,* Max T. Birch,* Simone Finizio, Lukas Powalla, Sayooj Satheesh, Tim Priessnitz, Eberhard Göring, Ernst Knöckl, Christoph Kastl, Alexander Holleitner, Klaus Kern, Markus Weigand, Sebastian Wintz, and Marko Burghard**

**Contents**

1. SQUID measurements of FGT bulk sample

2. Calculation of strain distribution from AFM height profile

3. Finite element simulation of strain distribution

4. Determination of local strain using Raman microscopy

5. X-ray absorption spectra and sum rules analysis

6. Correlation between out-of-plane magnetization and local strain

7. Extended STXM images for phase diagrams

8. Temperature dependent domain formation

9. Emergence of composite skyrmions

10. Optimal thickness of the FGT flakes on micropillars

**Supplementary Note 1: SQUID measurements of FGT bulk sample**

We measured magnetization as a function of temperature with a magnetic field of 300 Oe applied along both the out-of-plane (**Figure S1**a) and in-plane (Figure S1b) directions of the FGT crystal lattice. Each plot shows two measurement procedures: Field cooling (FC) from 300 K to 2 K in the presence of the field; and zero-field cooling (ZFC), i.e., warming at a fixed field from 2 K to 300 K after initial cooling in zero field. The extracted Curie temperature of approximately 210 K is consistent with previous reports.^[1,2]^

Magnetization as a function of applied magnetic field (μ_0_H) at 2 K is displayed in Figure S1c for both out-of-plane and in-plane field orientations. The hysteresis loops clearly demonstrate that the c-axis (out-of-plane direction) is the easy axis of magnetization.


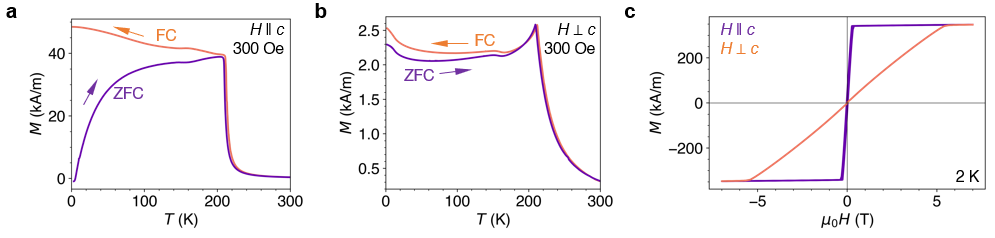


**Figure S1**. Magnetometry measurements of the bulk Fe_3_GeTe_2_ crystal. a,b) Magnetization vs. temperature measurements with the magnetic field aligned either parallel (H ‖ c) (a) or perpendicular (H ⊥ c) (b) to the c-axis of the bulk FGT crystal. Measurements were conducted using two methods: field cooling (FC, orange) from 300 K to 2 K, and zero field cooling (ZFC, purple), where the sample was initially cooled to 2 K without a magnetic field and then measured from 2 K to 300 K under a magnetic field of 300 Oe. c) Magnetization vs. field measurements at 2 K for both parallel (H ‖ c, purple) and perpendicular (H ⊥ c, orange) field alignments.

**Supplementary Note 2: Calculation of strain distribution from AFM height profile**

The induced local strain distribution is estimated using the strain tensor defined as:

$$\varepsilon_{ij}\left( \boldsymbol{r} \right)=\frac{1}{2}\left( \partial_{i}u_{j}\left( \boldsymbol{r} \right)+\partial_{j}u_{i}\left( \boldsymbol{r} \right)+\partial_{i}h\left( \boldsymbol{r} \right)\partial_{j}h\left( \boldsymbol{r} \right) \right),$$

where u(**r**) and h(**r**) represent the in-plane and out-of-plane deformation fields, respectively. This analysis relies upon the assumption that the in-plane lattice distortion is significantly smaller than the vertical lattice distortion.^[3]^ We focus on the deformation caused by the height of the Al micropillars (245 nm), taking the first two terms of the right-hand side to be zero. The in-plane strain components are therefore calculated from out-of-plane deformation field h(**r**) which is determined by atomic force microscopy (AFM):

$$\varepsilon_{xx}=\frac{1}{2}\left( \frac{\partial h(\boldsymbol{r})}{\partial x} \right)^{2},$$

$$\varepsilon_{yy}=\frac{1}{2}\left( \frac{\partial h(\boldsymbol{r})}{\partial y} \right)^{2},$$

$$\varepsilon_{xy}=\frac{1}{2}\frac{\partial h(\boldsymbol{r})}{\partial x}\frac{\partial h(\boldsymbol{r})}{\partial y},$$

The resulting uniaxial strain distributions $\varepsilon_{xx}$ and $\varepsilon_{yy}$ are presented in **Figure S3**a and Figure S3b, respectively. To align with features in the STXM image (**Figure S2**), we introduce the spatially resolved isotropic biaxial tensile $1/2(\varepsilon_{xx}+\varepsilon_{yy})$ (Figure S3c) and the antisymmetric shear strain $1/2(\varepsilon_{xx}-\varepsilon_{yy})$ (Figure S3d).^[4]^ It can be seen that $\varepsilon_{xy}$ qualitatively matches changes in magnetization profile visible in the STXM images in Figure S2, although it should be noted that at the corners both tensile and shear strain contribute to the total strain.


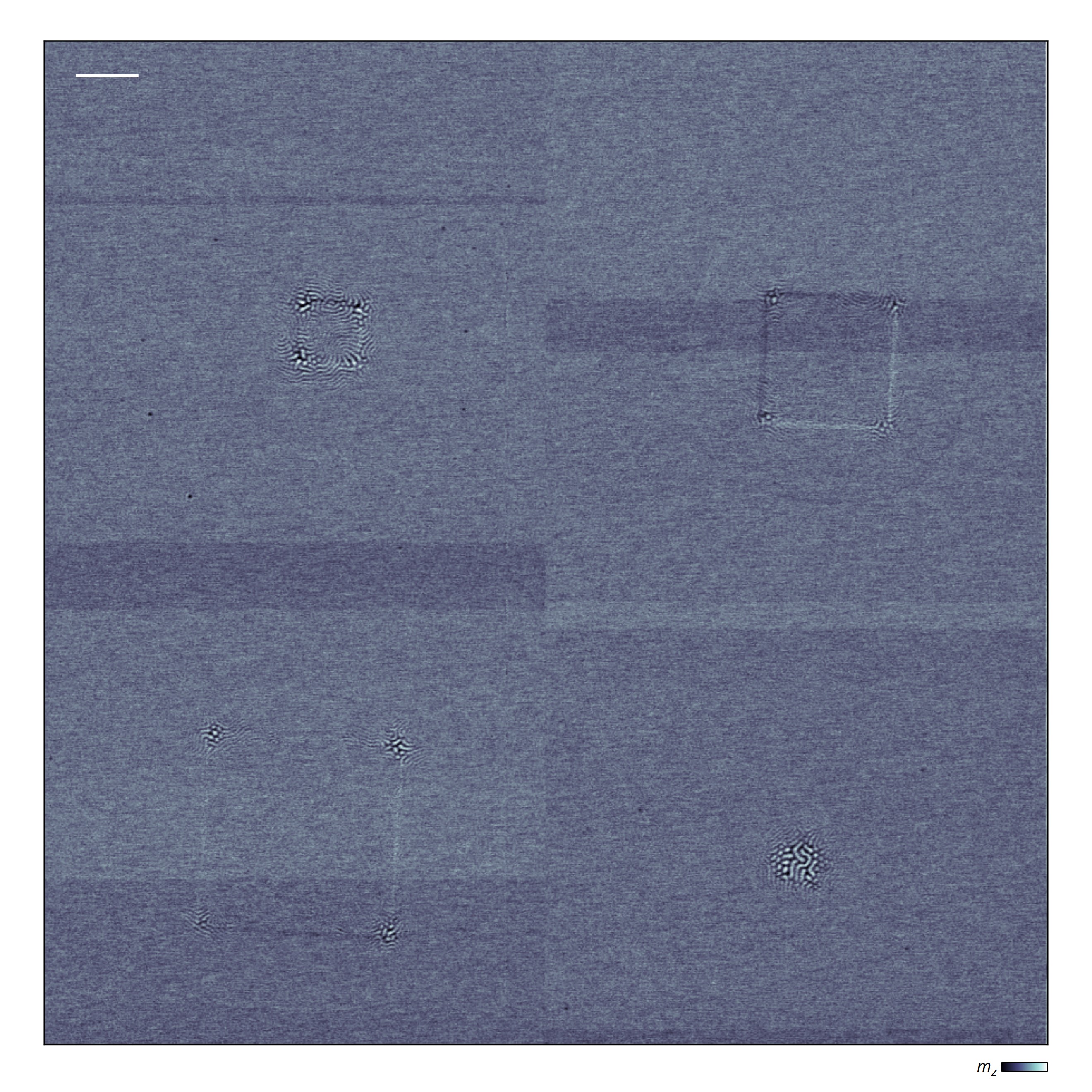


**Figure S2. STXM Image of region of interest (ROI) of the exfoliated Fe_3_GeTe_2_ flake.** This X-ray micrograph, acquired at 218 K and 0 mT, shows the ROI as defined in Figure 1d, featuring the formation of localized spin textures due to the strain induced by the four-square pillars with side lengths of 1, 2, 4, and 6 µm. The image is a subtraction of two images acquired for left and right circularly polarized light. Scale bar: 2 µm.


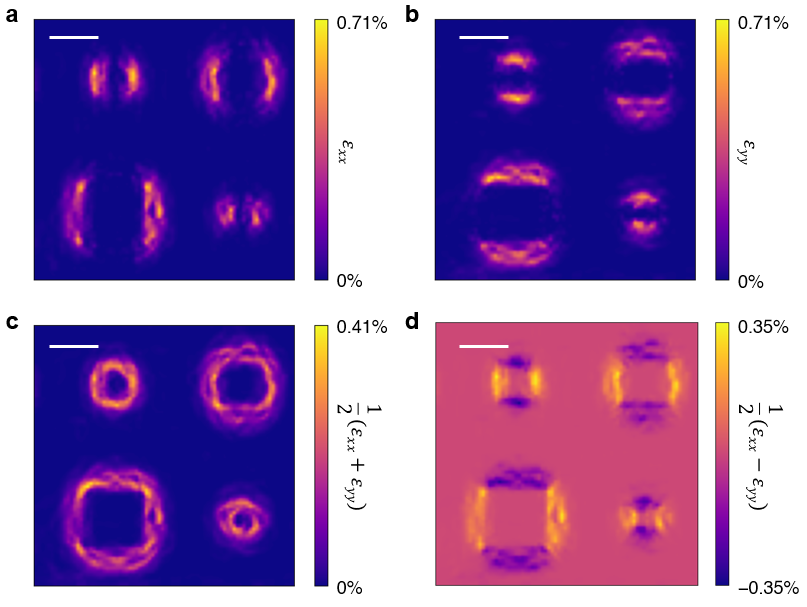


**Figure S3**. **Analysis of local strain distribution in the region of interest (ROI).** a,b) Calculated local uniaxial strain distribution along the x-axis (a) and y-axis (b), highlighting directional strain variations. c,d) Diagonalized strain distributions derived from the calculations in (a) and (b). Calculated biaxial tensile strain (c) and antisymmetric uniaxial strain (d), resulting from the differential uniaxial strains along the x and y directions, respectively. Scale bar: 5 µm.

**Supplementary Note 3: Finite element simulation of strain distribution**

To obtain the full mechanical strain information for the FGT sheet, we perform a numerical simulation of the region of interest (ROI) based on the finite element method (FEM) using COMSOL Multiphysics^®^ 6.1.^[5]^ We estimate the mechanical properties of FGT based on values reported for other common 2D materials,^[6]^ i.e. we use density $\rho=7300$ kg/m^3^, Young’s modulus $E=265$ GPa and Poisson’s ratio 0.25. The pillars have the same nominal dimensions as in the experiments, i.e., a height of 245 nm and varying edge width $w_{pillar}$ of 1 µm, 2 µm, 4 µm and 6 µm, respectively. To match the experimentally observed topography (see Figure 1h in the main text) of the FGT flake, we enforce the flake to touch the Si_3_N_4_ membrane outside of a region around a pillar given by a circle of radius ${r=w}_{pillar}+3.335$ µm centered on each pillar. We note that this assumption results in artifacts at the radial edges where contact to the membrane is enforced. Nevertheless, we find the simulation results (**Figure S4**) to be in good agreement with the strain values estimated based on the measured AFM topography (see Supplementary Note 2 and Figure 2 in the main text). While the magnitude of the strain may not be entirely quantitative, the results of the FEM simulations show strong qualitative agreement with the strain profiles obtained from the aforementioned AFM measurement.


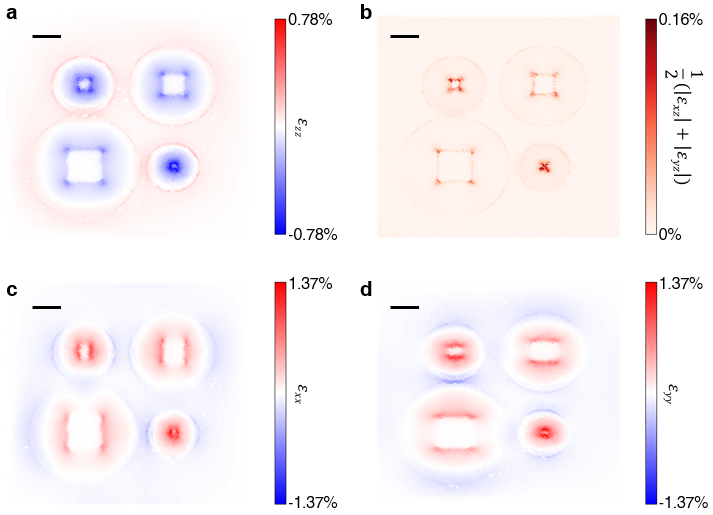


**Figure S4**. Simulation of local strain distribution in the region of interest (ROI): a,b) Simulated out-of-plane normal strain distribution (a) and shear strain distribution (b). c,d) Simulated in-plane uniaxial strain along x (c) and y (d) directions. Scale bar: 5 µm.

**Supplementary Note 4: Determination of local strain using Raman microscopy**

To independently verify the presence of strain in the FGT film on micropillars and to estimate its magnitude, we use Raman spectroscopy (**Figure S5**). A recent report demonstrated a red (blue) shift of the characteristic E^2^_2g_ (~126 cm^-1^) mode in FGT due to tensile (compressive) strain of about (1 cm^-1^)/(1% strain).^[7]^ All measurements are carried out on a WITec Alpha Raman microscope (532 nm cw-excitation, 55 µW laser power measured at the back aperture of the objective, 50x objective with NA = 0.75, 1800 lines/mm grating). All depicted error bars are the 1σ-confidence intervals of the parameter as evaluated by the fit routine.

Figure S5a depicts spectra when scanning across FGT on a pillar. The black spectra are taken at the center of the pillar and off the pillar, respectively. The red spectrum is taken from the edge. The characteristic E^2^_2g_ (~126 cm^-1^) and A^1^_1g_ (~144 cm^-1^) modes of FGT are clearly resolved in agreement with previous reports.^[7,8]^ The dashed line in Figure S5a serves as a guide to the eye, and it reveals a red shift of the red spectrum already in the raw data. The position of the E^2^_2g_ (~126 cm^-1^), when scanning across the pillar edge, is evaluated in Figure S5b. Close to the edges, a systematic red-shift of about 0.5 cm^-1^ is revealed with respect to the center of the pillar. The assigned edge position (red shaded areas) was independently verified from the optical microscope image. The observed red-shift of the E^2^_2g_ mode would be consistent with a tensile strain of about 0.5% within FGT regions close to the edges. The A^1^_1g_ mode is not expected to show a strain dependence within the resolution of our experiment.^[8]^

Figures S5c-e show the used fit model, which reproduces the data very well as evidenced by the randomly distributed residual. To account for asymmetric line shapes, we use a Breit-Wigner-Fano resonance model.^[9]^ In addition to the E^2^_2g_ and A^1^_1g_ modes, we consider a peak shoulder around 160 cm^-1^.


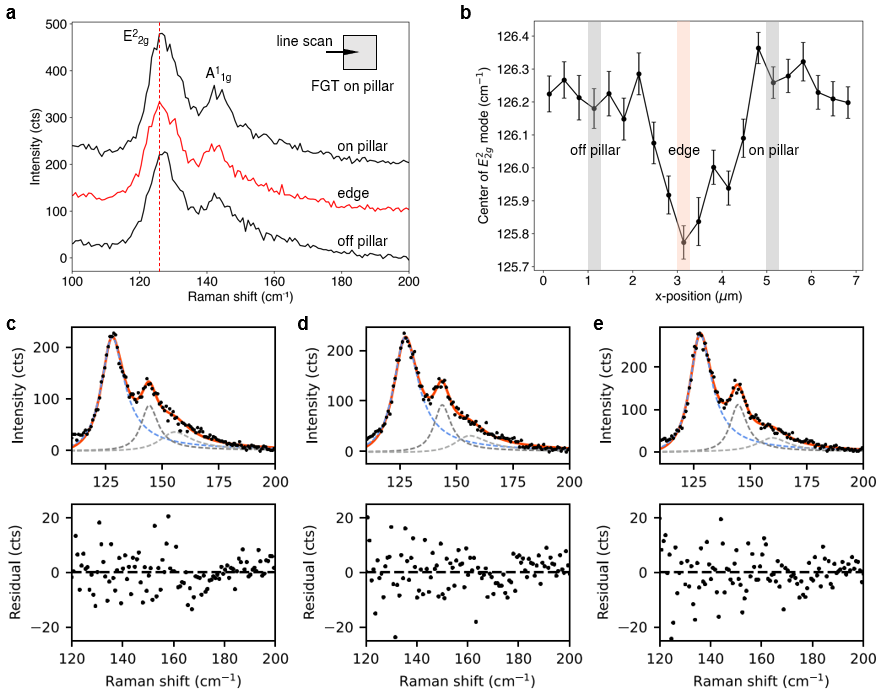


**Figure S5**. Identification of strain in the FGT film on micropillars via Raman microscopy. a) Raman spectra of FGT scanned across a square-shaped micropillar (inset). The red-shift of the modes can be discerned already in the bare spectrum (dashed line as guide to the eye). b) Fitted position of the E^2^_2g_ mode in FGT. c-e) Fit model and residual for the data shown in (a).

**Supplementary note 5: X-ray absorption spectra and sum rules analysis**

Soft X-ray excitations have the advantage of providing large resonances due to dipole-allowed transitions directly into the magnetic valence state.^[13]^ This includes the important 3*d* transition metal L_2,3_ (2*p*→3*d*) absorption edges.

Two XAS spectra were obtained at Fe L_2,3_ edges with right circular polarization for oppositely applied out-of-plane magnetic fields (**Figure S6**a). It is noteworthy that two distinct peaks are evident in the Fe L_3_ absorption edge, which can be attributed to the two distinct Fe ion species present in the FGT structure (designated as the Fe I and Fe II sites).^[1]^ The spectra were processed in the following manner: firstly, they were divided by I_0_ (the measured intensity of the synchrotron beam at that energy) and the logarithm base 10 was taken; secondly, a background was subtracted by performing a linear fit to the first 5 eV of the data set; and finally, the spectra were edge-normalized by dividing to set the after-edge value to unity.

X-ray magnetic circular dichroism (XMCD) is a phenomenon that arises from the breaking of time-reversal symmetry by a magnetic field. It is obtained by subtracting two X-ray absorption spectroscopy (XAS) spectra with circular polarization vectors parallel and antiparallel to the external magnetic field.^[13]^ The obtained integrated XMCD spectrum is presented in Figure S6b.

Figure S6c shows the ad hoc step function used to subtract the background from excitations into continuum states. The height of the steps at the L_3_ and L_2_ edges was set at a ratio of 2:1, in accordance with the occupation of the 2p_3/2_ and 2p_1/2_ core states.^[13]^ Once the non-resonant background had been subtracted, the resonant contribution of XAS and its integrated value were plotted in Figure S6d. In order to perform the sum rule analysis, orbital (m_L_) and spin (m_S_) magnetic moment are expressed as follows:

$$m_{L}=-\frac{4}{3}\frac{q}{r}n_{h},$$

$$m_{S}=-\frac{6p-4q}{r}n_{h},$$

where the number of holes n_h_ can be estimated using, for example, band structure calculation.^[14]^ In the case of FGT, n_h_ is taken to be 4. The orbital and spin contributions of the magnetic moment can thus be determined as 0.18 $\mu_{B}$ and 1.11 $\mu_{B}$ respectively, which are comparable to the results previously obtained for FGT.^[1]^ These values are the average through the thickness of the flake. It is likely slightly lower than the bulk value due to the presence of oxidized top and bottom layers, which thus contribute to the total absorption, but not the XMCD signal.


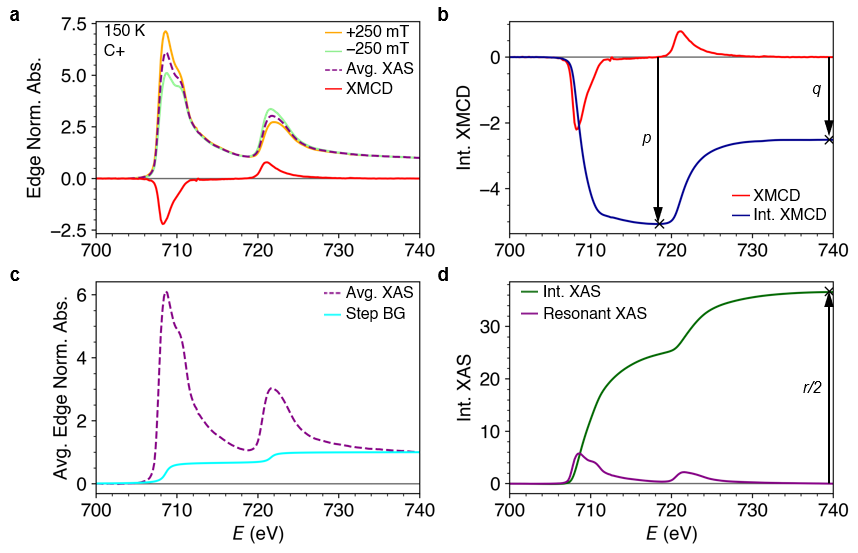


**Figure S6**. **X-ray Absorption spectra (XAS) of the Fe_3_GeTe_3_ flake measured in transmission.** a) Edge-normalized x-ray absorption spectra of the FGT flake at 150 K using right circular polarization under out-of-plane magnetic fields of ±250 mT. The difference between the spectra provides the x-ray magnetic circular dichroism (XMCD) signal. b) XMCD spectrum (red) and the integrated XMCD signal (dark blue). c) Average edge-normalized XAS (dashed purple) fitted with a double-step function (light blue) in order to extract the resonant contributions. d) Integrated XAS after subtraction of the background to obtain the resonant contributions. The integrated values in (b) and (d) were used in the subsequent sum rules analysis.

**Supplementary Note 6: Correlation between out-of-plane magnetization and local strain**

**Figure S7** features a line profile (panel b) that correlates the out-of-plane magnetization component *m*_z_​ at 230 K with local strain. Notably, for both in-plane tensile and in-plane shear strain, *m*_z_​ exhibits an approximately linear dependence on strain magnitude (panels e and f), suggesting that the strain effect does not exhibit a threshold behavior but rather scales continuously with the strain magnitude. This behavior is consistent with prior experimental and theoretical studies reporting an approximately linear relationship between *T*c​ and strain.^[10–12]^


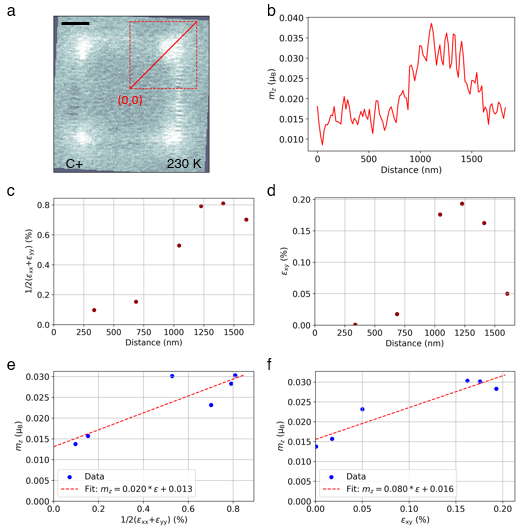


**Figure S7.** Correlation between the out-of-plane magnetization *m*_z_​ at 230 K and local strain components. a) STXM image of the FGT flake on top of the pillar with side length of 2 µm, acquired at C+ and 230 K. Scale bar: 500 nm. b) Profile of *m*_z_, taken along the red solid line in b), with the origin defined at the center of the pillar. c,d) Corresponding line profiles of in-plane tensile (c) and shear (d) strain components. e,f) Correlation plots of m_z_ with in-plane tensile (e) and shear (f) strain, demonstrating a linear relationship.

**Supplementary note 7: Extended STXM images for phase diagrams**

We performed STXM imaging using the field sweep (FS) protocol to map out the phase diagrams presented in Figure 5a-c. The FS series of STXM images at four temperatures lend support to the hypothesis that the influence of the strain depends sensitively on the sample temperature. In the data sets acquired at 150 K (**Figure S8**a-d) and 210 K (Figure S8e-h), the characteristic domain sizes are too large to allow observation of localized spin texture variation. As temperature is increased to 213 K (Figure S8i-l), the domain size decreases to a value larger than that of the micropillar (~4 µm). This results in the observation of a distinct region above and around the pillar with spin textures that differ from those at the edge of imaging area. Upon reaching a temperature of 227 K (Figure S8m-p), the domain size decreases to a value comparable to that of the corner of the micropillar (~500 nm). Localized spin textures were observed exclusively at the four corners of the micropillar.


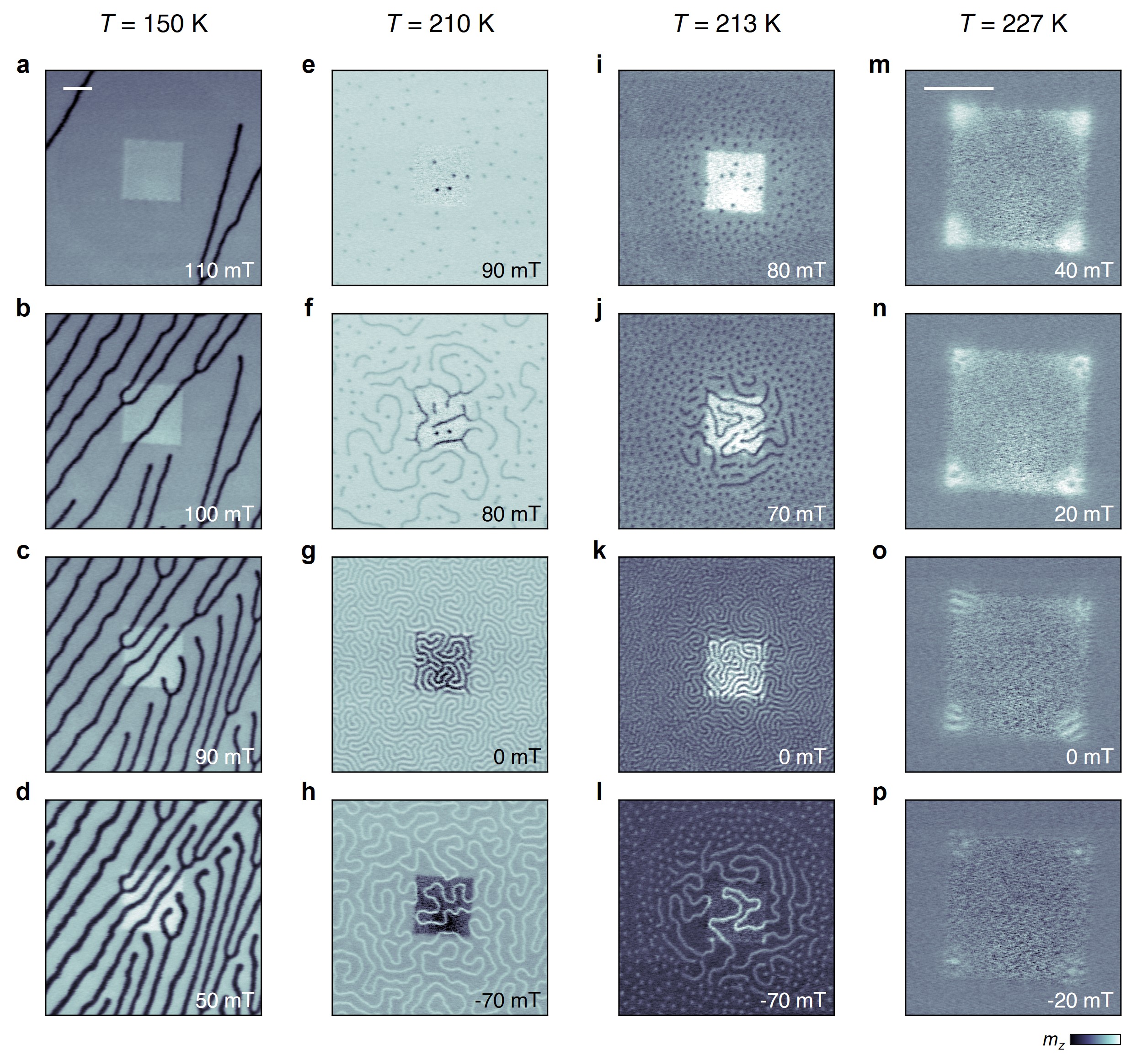


**Figure S8**. **Additional data sets for field sweep x-ray microscopy measurements. a-p)** X-ray micrographs of the FGT flake on top of the Al pillar with a side length of 2 µm, acquired following the field sweep procedure, at temperatures of 150 K (a-d), 210 K (e-h), 213 K (i-l), and 227 K (m-p). Scale bar: 1 µm.

**Supplementary note 8: Temperature dependent domain formation**

**Figure S9** compares the magnetic textures that are formed during the field sweep protocol at a constant out-of-plane magnetic field for varying temperatures. In each case, the FGT flake was initially prepared in a saturated state at −250 mT. At $\mu_{0}H=50 mT$ (Figure S9a-e the FGT flake undergoes a transition from a stripe domain state to a labyrinthine domain state with a more complex, maze-like pattern as temperature increases, which is consistent with previous reports of FGT.^[1,2]^ The real-space STXM measurements also demonstrate that the average domain size is increased at lower temperatures. This result is quantified in Figure S9f, which plots the average domain size at 0 mT as a function of temperature. At $\mu_{0}H=90 mT$ (Figure S9g-j), an unusual domain state with a radially symmetric pattern, centered on the pillar (Figure S9j), was observed.


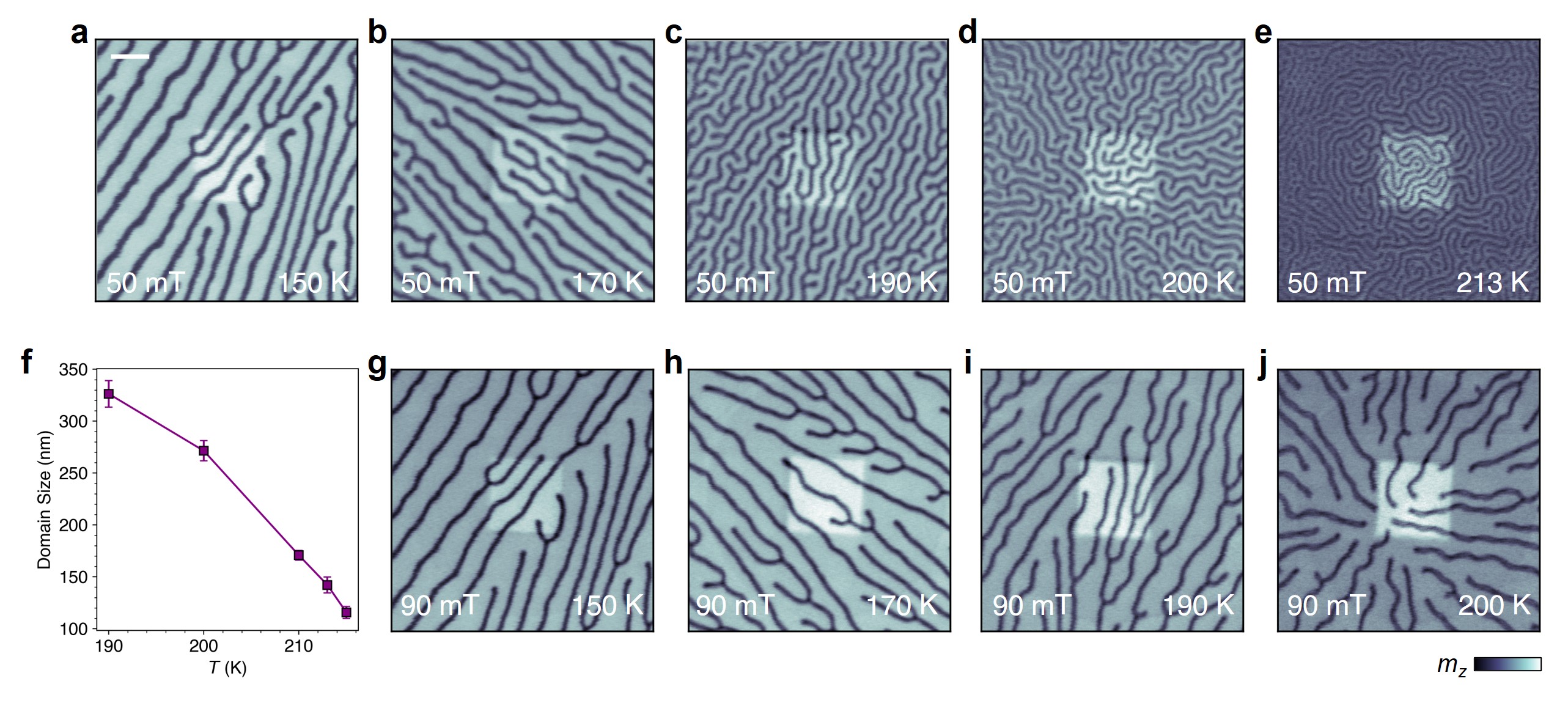


**Figure S9**. Temperature-dependent domain formation. X-ray micrographs showing domain formation during the field sweep protocol at an out-of-plane magnetic field of 50 mT (a-e) and 90 mT (g-j). f) Temperature dependence of domain size measured at 0 mT. Scale bar: 1 µm.

**Supplementary note 9: Emergence of composite skyrmions**

In **Figures S10-11**, we present field sweeps of STXM images taken at specific temperatures (215-218 K) for the FGT flake strained by differently sized micropillars (1, 4, 6 µm). As observed in the results of the 2 μm micropillar presented in the main text, the formation of composite skyrmions, skyrmioniums and skyrmion bags, is also evident. These structures form locally at the corners of pillars, which correspond to the regions of high strain. The conclusions drawn from the data set of the 2 µm micropillar can be transferred to cases involving differently sized pillars, and confirm that the local strain profile is responsible for the phenomena.


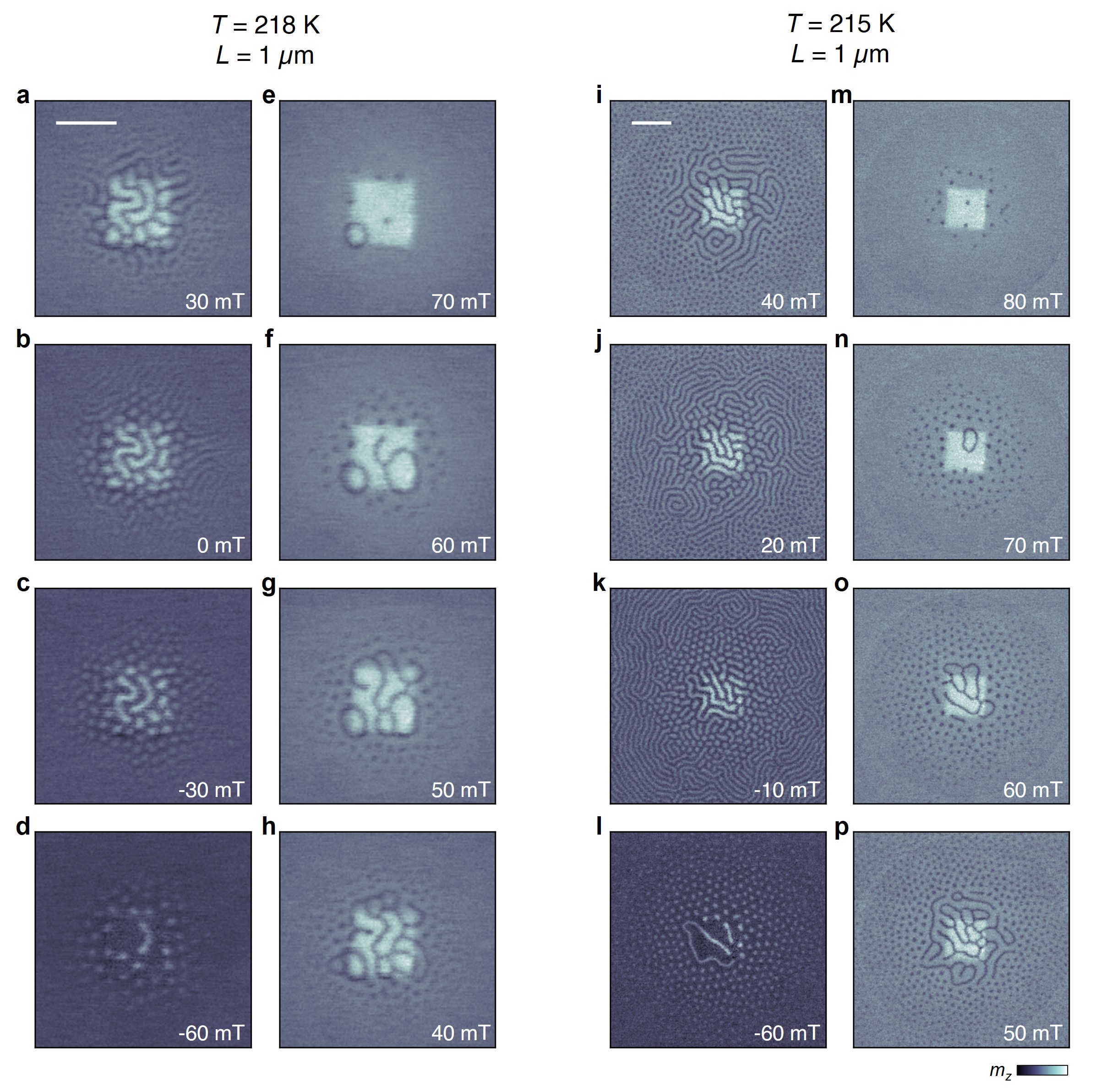


**Figure S10**. **Formation of composite skyrmion states at the corners of the micropillars.** X-ray micrographs of the FGT flake on top of the aluminum pillar with 1 µm side length, acquired following the field sweep procedure, at temperatures of 218 K (a-h) and 215 K (i-p). Skyrmionium formation is observed in (e), (f), (g), and (n), while a skyrmion bag is seen in (h). Scale bar: 1 µm.


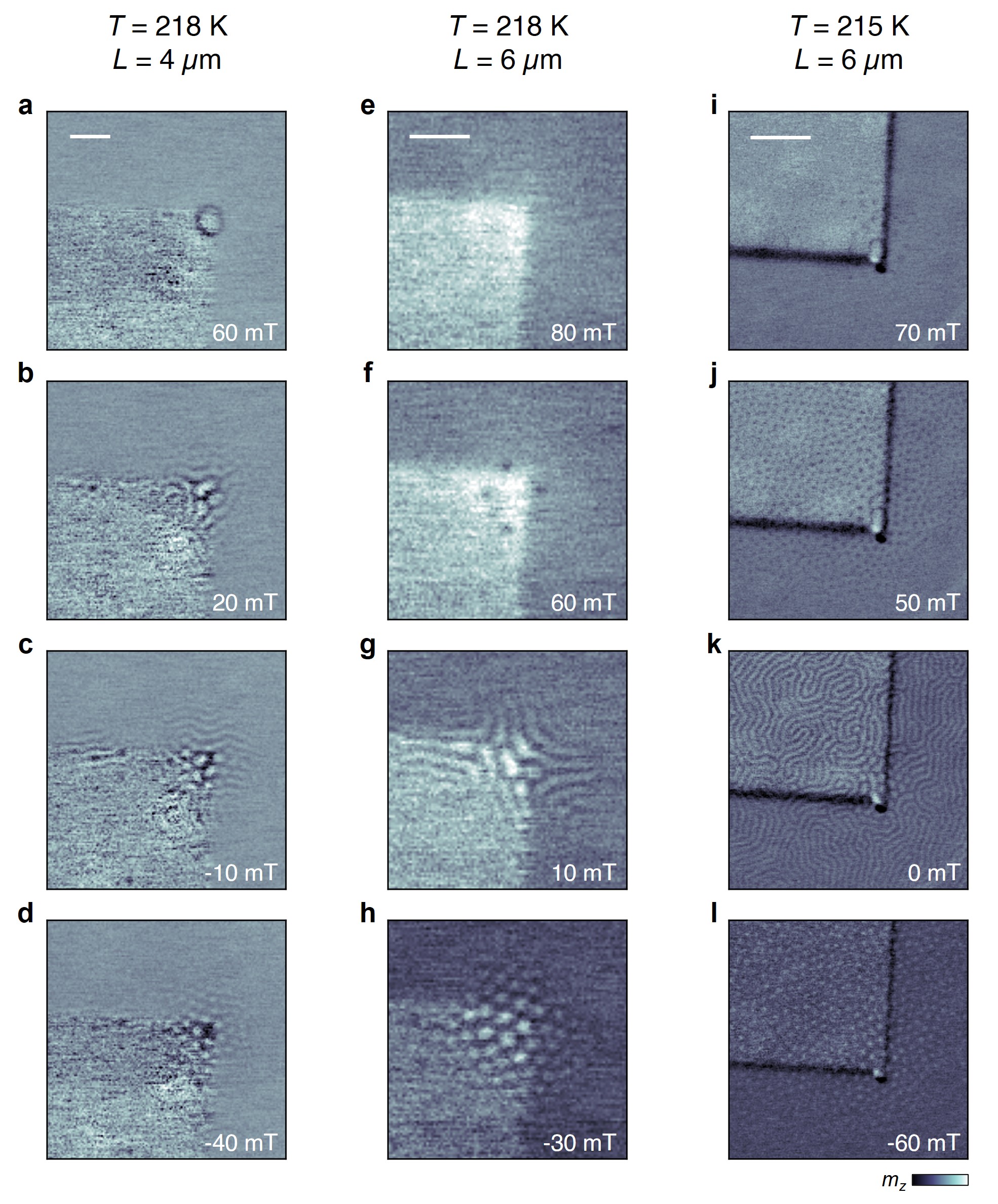


**Figure S11**. Example of **field sweep data for pillars of different sizes.** X-ray micrographs showing the results of field sweeps on pillars with side lengths of 4 µm (a-d) and 6 µm (e-l) at temperatures of 218 K (a-h) and 215 K (i-l). The emergence of skyrmionium is observed in (a) and (i), while a skyrmion bag appears in (j). Scale bar: 500 nm.

**Supplementary Note 10: Optimal thickness of the FGT flakes on micropillars**

In our experiments, the thinnest flakes we were able to transfer onto the micropillar arrays were 16 nm thick. Notably, FGT flakes with thicknesses in the range of 10 - 20 nm tend to form narrow, corner-localized wrinkles and exhibit pronounced bending over the pillar edges. In contrast, thicker flakes deform more smoothly over the micropillars, as shown in **Figure S12**a.

In general, we faced the following difficulties while studying thinner FGT flakes:

1. The corner wrinkles in thin flakes are not reproducible. As illustrated in Figure S12a, the deformation varies from corner to corner, primarily due to the reduced stiffness of thinner flakes. This results in significant variation in local strain, not only between samples but even within a single flake. In comparison, deformation in a 135 nm-thick flake is highly consistent and reproducible (see Figures S12b-c).
2. For flakes thinner than ~20 nm, surface oxidation becomes a serious concern, necessitating encapsulation with hBN. However, hBN capping substantially alters the deformation behavior on micropillars, making it difficult to extract accurate strain maps. Therefore, our measurements were performed without this capping, with care taken to minimize the exposure to the air.
3. As seen in Figures S12d-e, thin flakes are more susceptible to mechanical damage during stamping, often resulting in the puncturing, tearing or fracturing around the corners.

For the thicker flakes, the primary limitation is the absorption of soft x-rays, which cannot penetrate FGT flakes thicker than ~300 nm according to our previous experience on FGT. Taken together, we find that flakes around 150 nm in thickness offer an optimal balance for our study, providing sufficient structural integrity and strain without exceeding the soft x-ray penetration depth.


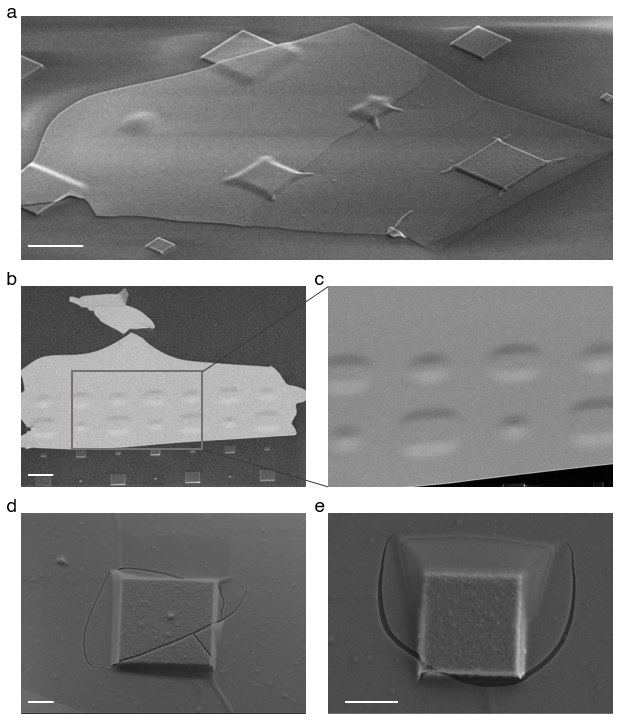


**Figure S12**. a) Direct comparison of the deformation behavior over micropillars between thin and thick regions of FGT. Scale bar: 5 µm. b,c) SEM images of a thick flake (135 nm) stamped onto a micropillar array, displaying the smooth deformation over all pillars. Scale bar: 10 µm. d,e) SEM images of thin flakes with thicknesses of 18 nm (d) and 16 nm (e) on micropillars, exhibiting narrow, corner-localized wrinkles and the presence of cracks at distinct locations. Scale bar: 1 µm.

**References**

[1] M. T. Birch, L. Powalla, S. Wintz, O. Hovorka, K. Litzius, J. C. Loudon, L. A. Turnbull, V. Nehruji, K. Son, C. Bubeck, T. G. Rauch, M. Weigand, E. Goering, M. Burghard, G. Schütz, *Nat. Commun.* **2022**, *13*, 3035

[2] M. T. Birch, L. Powalla, K. Litzius, V. Nehruji, O. Hovorka, S. Wintz, F. Schulz, D. A. Mayoh, G. Balakrishnan, M. Weigand, M. Burghard, G. Schütz, *2D Mater.* **2024**, *11*, 025008

[3] D.-H. Kang, H. Sun, M. Luo, K. Lu, M. Chen, Y. Kim, Y. Jung, X. Gao, S. J. Parluhutan, J. Ge, S. W. Koh, D. Giovanni, T. C. Sum, Q. J. Wang, H. Li, D. Nam, *Nat. Commun.* **2021**, *12*, 5087

[4] D. Walkup, B. A. Assaf, K. L. Scipioni, R. Sankar, F. Chou, G. Chang, H. Lin, I. Zeljkovic, V. Madhavan, *Nat. Commun.* **2018**, *9*, 1550

[5] COMSOL Multiphysics® v. 6.1. www.comsol.com. COMSOL AB, Stockholm, Sweden

[6] Y. Li, C. Yu, Y. Gan, P. Jiang, J. Yu, Y. Ou, D.-F. Zou, C. Huang, J. Wang, T. Jia, Q. Luo, X.-F. Yu, H. Zhao, C.-F. Gao, J. Li, *Npj Comput. Mater.* **2018**, *4*, 1

[7] L. Hu, J. Zhou, Z. Hou, W. Su, B. Yang, L. Li, M. Yan, *Mater. Horiz.* **2021**, *8*, 3306

[8] L. Hu, F. Liu, Q. Quan, C. Lu, S. Yu, L. Li, *Adv. Funct. Mater.* **2024**, *34*, 2409085

[9] E. H. Hasdeo, A. R. T. Nugraha, M. S. Dresselhaus, R. Saito, *Phys. Rev. B* **2014**, *90*, 245140

[10] H. Ren, G. Xiang, *Nanomaterials* **2023**, *13*, 2378

[11] R. Fujita, G. Gurung, M.-A. Mawass, A. Smekhova, F. Kronast, A. K.-J. Toh, A. Soumyanarayanan, P. Ho, A. Singh, E. Heppell, D. Backes, F. Maccherozzi, K. Watanabe, T. Taniguchi, D. A. Mayoh, G. Balakrishnan, G. van der Laan, T. Hesjedal, *Adv. Funct. Mater.* **2024**, *34*, 2400552

[12] Y. Wang, C. Wang, S.-J. Liang, Z. Ma, K. Xu, X. Liu, L. Zhang, A. S. Admasu, S.-W. Cheong, L. Wang, M. Chen, Z. Liu, B. Cheng, W. Ji, F. Miao, *Adv. Mater.* **2020**, *32*, 2004533

[13] G. Van Der Laan, A. I. Figueroa, *Coord. Chem. Rev.* **2014**, *277–278*, 95

[14] C. T. Chen, Y. U. Idzerda, H.-J. Lin, N. V. Smith, G. Meigs, E. Chaban, G. H. Ho, E. Pellegrin, F. Sette, *Phys. Rev. Lett.* **1995**, *75*, 152
